# DFNB1 Non-syndromic Hearing Impairment: Diversity of Mutations and Associated Phenotypes

**DOI:** 10.3389/fnmol.2017.00428

**Published:** 2017-12-22

**Authors:** Francisco J. del Castillo, Ignacio del Castillo

**Affiliations:** ^1^Servicio de Genética, Hospital Universitario Ramón y Cajal, IRYCIS, Madrid, Spain; ^2^Centro de Investigación Biomédica en Red de Enfermedades Raras (CIBERER), Madrid, Spain

**Keywords:** hearing impairment, inner ear, DFNB1, GJB2, connexin-26, GJB6, connexin-30

## Abstract

The inner ear is a very complex sensory organ whose development and function depend on finely balanced interactions among diverse cell types. The many different kinds of inner ear supporting cells play the essential roles of providing physical and physiological support to sensory hair cells and of maintaining cochlear homeostasis. Appropriately enough, the gene most commonly mutated among subjects with hereditary hearing impairment (HI), *GJB2*, encodes the connexin-26 (Cx26) gap-junction channel protein that underlies both intercellular communication among supporting cells and homeostasis of the cochlear fluids, endolymph and perilymph. *GJB2* lies at the DFNB1 locus on 13q12. The specific kind of HI associated with this locus is caused by recessively-inherited mutations that inactivate the two alleles of the *GJB2* gene, either in homozygous or compound heterozygous states. We describe the many diverse classes of genetic alterations that result in DFNB1 HI, such as large deletions that either destroy the *GJB2* gene or remove a regulatory element essential for *GJB2* expression, point mutations that interfere with promoter function or splicing, and small insertions or deletions and nucleotide substitutions that target the *GJB2* coding sequence. We focus on how these alterations disrupt *GJB2* and Cx26 functions and on their different effects on cochlear development and physiology. We finally discuss the diversity of clinical features of DFNB1 HI as regards severity, age of onset, inner ear malformations and vestibular dysfunction, highlighting the areas where future research should be concentrated.

## Introduction

Hearing is an extraordinarily complex process that involves many cell types and diverse cellular and molecular structures and mechanisms. The genetic causes of hereditary hearing impairment (HI) are, in consequence, very heterogeneous. Over one hundred genes underlying nonsyndromic hearing impairment (NSHI) have been identified so far^1^ and it is estimated that many more genes remain to be identified. Unsurprisingly, most of these genes encode proteins that participate in different aspects of the physiology of the cochlear sensory hair cells, including auditory mechanotransduction and synaptic transmission mechanisms (Richardson et al., 2011; Safieddine et al., 2012). Yet, the gene most frequently mutated among subjects with NSHI in many populations is not at all expressed in hair cells. This gene, *GJB2*, lies at the DFNB1 locus on chromosome 13q12, and codes for connexin-26 (Cx26), a gap junction protein that is essential for the function of cochlear supporting cells.

DFNB1 owes its name to the first mapped locus for nonsyndromic deafness (DFN) with autosomal recessive inheritance (B; Guilford et al., 1994), and the acronym has eventually come to represent the specific type of HI caused by mutations at this locus (MIM# 220290). The identification of the first *GJB2* mutations causing DFNB1 HI (Kelsell et al., 1997) was soon followed by screenings that revealed a high frequency of *GJB2* mutations among subjects with NSHI (Denoyelle et al., 1997; Zelante et al., 1997). Since then, high prevalence of DFNB1 HI was demonstrated in most populations (Chan and Chang, 2014), and so searching for mutations affecting *GJB2* quickly became the mainstay for the genetic diagnosis of NSHI. The interest on DFNB1 HI spurred many diverse studies to explore the underlying pathogenic mechanisms, ranging from functional assays of wild-type and mutant proteins to generation and analysis of DFNB1 mouse models. Here we review our current knowledge on the molecular pathology and clinical features of DFNB1 HI.

## Genetic Alterations That Result in DFNB1 Hearing Impairment

*GJB2* is 5513 bp long and contains two exons (193 bp and 2141 bp long, respectively) separated by a 3179-bp intron (Kiang et al., 1997). Transcription is initiated from a single start site and leads to the synthesis of a 2334-nucleotide mRNA (GenBank NM_004004.5), which is considered canonical. The 678-bp sequence that codes for Cx26 is completely contained within *GJB2* exon 2. A genome-wide search for alternative transcriptional start sites found an expressed sequence tag (EST) that suggested the existence of an alternative 184-bp first exon, located within the only *GJB2* intron (Kimura et al., 2006), which would lead to the production of a 2318-nucleotide mRNA (GenBank XM_011535049.2). This EST (GenBank DA975033.1) was found in a cDNA library of synovial membrane tissue from rheumatoid arthritis patients. Further research is needed to determine whether this mRNA is expressed in the inner ear, a relevant issue given that this alternative first exon could be a novel target for genetic screening in patients with NSHI (Parzefall et al., 2017).

Cx26 belongs to the connexin family of integral membrane proteins, which act as subunits of a hexameric annular assembly, termed connexon, which forms a pore through the lipid bilayer. Connexons can be composed by connexins of either the same or different type (homo- or heteromeric connexons, respectively; Kumar and Gilula, 1996). Because of this combinatorial capability, pathogenic mutations in connexin genes can produce a variety of clinical outcomes, and so it occurs to *GJB2*. Some missense mutations, inherited in a dominant form, result in Cx26 mutant subunits that exert dominant negative effects on the activity of wild-type Cx26 alone or on Cx26 and connexin-30 (Cx30) together. They cause the DFNA3 type of autosomal dominant nonsyndromic HI (NSHI; Denoyelle et al., 1998; Forge et al., 2003b; Marziano et al., 2003; Zhang et al., 2011). Other dominant missense mutations result in Cx26 mutant subunits that impact the activity of both wild-type Cx26 and connexin-43 (Rouan et al., 2001). They cause different syndromes associating HI with skin disorders, such as Vohwinkel (Maestrini et al., 1999) or Bart-Pumphrey (Richard et al., 2004) syndromes, keratitis-ichthyosis-deafness (KID; Richard et al., 2002; van Steensel et al., 2002), hystrix-like ichthyosis and deafness (HID; van Geel et al., 2002), palmoplantar keratoderma with deafness (PPK-D; Richard et al., 1998) or sensorineural deafness with erythematous plaques, cutaneous orthokeratotic hyperkeratosis and parakeratosis in the oral and esophageal mucosa (Brown et al., 2003).

However, a majority of *GJB2* mutations are recessively inherited, and result in DFNB1 NSHI when they are in homozygous or compound heterozygous states (Kelsell et al., 1997). About 200 *GJB2* mutations have been reported to cause DFNB1 HI^2^ (Stenson et al., 2017). They can be classified into many different types: large deletions that remove the whole *GJB2* gene (Feldmann et al., 2009; Bliznetz et al., 2014, 2017), large deletions that remove regulatory sequences that are needed for the expression of *GJB2* but keep the gene intact (del Castillo et al., 2002, 2005; Wilch et al., 2010; Tayoun et al., 2016; Table 1), and a plethora of small-scale alterations, including nonsense, missense and splice-site point mutations, as well as frameshifting small insertions and deletions (del Castillo and del Castillo, 2011). The frequencies of these mutations are diverse, with different mutant alleles overrepresented in different populations. The genetic epidemiology of DFNB1 HI has been extensively reviewed (Chan and Chang, 2014), and so we will focus on the molecular mechanisms by which these mutations alter the functions of the *GJB2* gene and the Cx26 protein.

**Table 1:** Large deletions at the DFNB1 locus on 13q12.

Abbreviated name	Name including exact coordinates^1^	Size of deleted interval	Reference
del(*GJB6*-D13S1830)	Chr13:g.(20,797,177_21,105,945)del	309 kb	del Castillo et al. (2002)
del(*GJB6*-D13S1854)	Chr13:g.(20,802,727_21,034,768)del	232 kb	del Castillo et al. (2005)
del(131-kb)	Chr13:g.(20,939,344_21,070,698)del	131 kb	Wilch et al. (2010)
del(179-kb)	Chr13:g.(20,921,711_21,101,115)del	179 kb	Tayoun et al. (2016)
del(920-kb)	Not applicable	>920 kb	Feldmann et al. (2009)
del(101-kb)	Chr13:g.(20,757,021_20,858,394)del	101 kb	Bliznetz et al. (2014)

*^1^First and last nucleotide that are eliminated by the deletion, Human Genome Build GRCh37/hg19*.

### Mutations That Alter *GJB2* Expression

**Figure 1:**
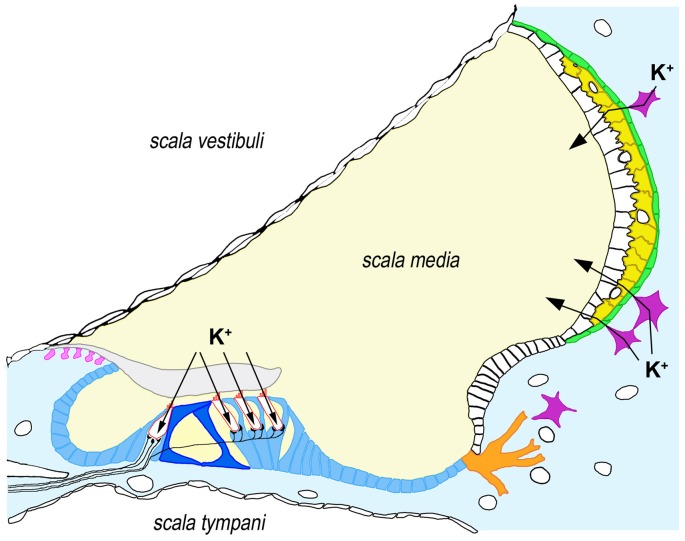
Expression of connexin-26 (Cx26) and connexin-30 (Cx30) in the human cochlea. Cx26 and Cx30 are synthesized by all supporting cells types within the organ of Corti (blue), including inner and outer pillar cells (dark blue), as well as by root cells (orange), interdental cells (pink), fibrocytes from the underlying connective tissue (light blue) and basal (green) and intermediate cells (yellow) from the stria vascularis. For the sake of clarity, we have depicted only four fibrocytes (purple) in connective tissue. Arrows indicate the pathways for K^+^ influx in hair cells (bordered in red) and K^+^ secretion into endolymph through the stria vascularis. It must be noted that Cx26 and Cx30 seem to form distinct, homomeric plaques even when co-expressed in the same cell, although these plaques are closely associated. Some immuhistochemical staining experiments suggest that co-expression may not occur in all the cell types indicated above (e.g., Cx26 may not be synthesized in inner pillar cells and strial intermediate cells, whereas Cx30 may not be synthesized in strial basal cells), which might reflect specific functional associations with additional ion transporters (Liu et al., 2009, 2016).

*GJB2* expression takes place in specific cell types in many different tissues. With the significant exception of hair cells, *GJB2* is expressed by nearly all cell types within the human cochlea, including supporting cells in the sensory epithelium, fibrocytes and mesenchymal cells in the lateral wall, basal and intermediate cells of the stria vascularis and type I neurons in the spiral ganglion (Figure 1; Liu et al., 2009).

Interestingly, a study performed on the closely related murine *Gjb2* promoter (81% sequence identity) revealed that *Gjb2* and the neighboring *Gjb6* gene (encoding mouse Cx30) are transcriptionally co-regulated in cochlear supporting cells upon activation of NF-κB (Ortolano et al., 2008), which is likely caused by intracellular Ca^2+^ oscillations induced by connexin signaling (see “Mutations Affecting the Function of Cx26 Hemichannels” section below; Rodriguez et al., 2012). This mechanism may be not directly extrapolatable to the expression of the human ortologs, since the murine promoter contains an NF-κB binding site that is absent in the human promoter. Further studies are needed to investigate a putative co-regulation of *GJB2* and *GJB6* in human cochlea.

#### Mutations Affecting the *GJB2* Promoter

**Figure 2:**
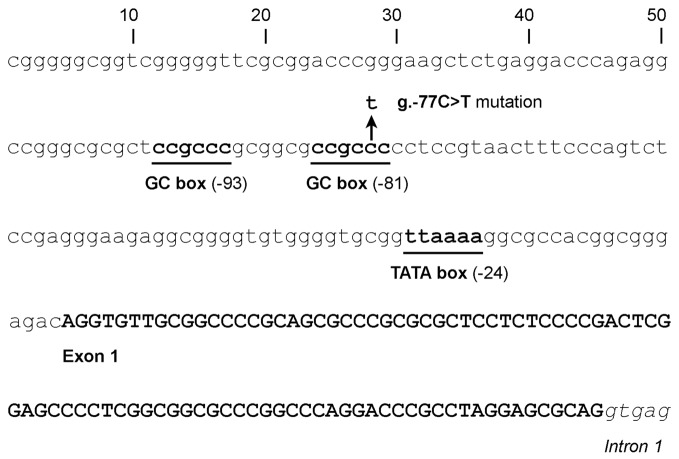
Sequence of the *GJB2* basal promoter and first exon, showing the location of the GC boxes (at -81 and -93), the TATA motif (at -24) and the transcription start site of the first canonical exon. The arrow indicates the position of the g.-77C>T promoter mutation.

The 128-bp-long basal promoter of *GJB2* lies just upstream of the canonical first exon (Kiang et al., 1997; Tu and Kiang, 1998). The promoter includes a TATA box and two GC boxes (Figure 2), which are bound by the Sp1 and Sp3 transcription factors, as demonstrated by *in vitro* experiments and by the fact that the promoter activity is drastically reduced by engineered mutations in either of the GC boxes (Tu and Kiang, 1998).

So far only one human mutation has been reported to affect the *GJB2* promoter. It was found in the compound heterozygous state with mutation p.(Val84Met) in a Portuguese hearing-impaired subject (Matos et al., 2007). This promoter mutation, g.-77C>T (also known as -3438C>T and c.-259C>T), affects the GC box at -81 (CCGCCC > CCGCTC). Taking into account the effects of the *in vitro* engineered mutations in the GC boxes, it was expected that this naturally occurring mutation would impair the binding of Sp1 and Sp3. In fact, the mutant promoter exhibited a drastically reduced activity in reporter-gene experiments performed in different cell lines (Matos et al., 2007).

#### Deletions Removing a Distal Enhancer

When mutation screening of the *GJB2* coding region in subjects with autosomal recessive NSHI became a general practice, it was soon evident that there was an overrepresentation of affected subjects in whom only one heterozygous pathogenic mutation could be found. It was hypothesized that the missing mutations might affect non-coding regulatory sequences or other genes. Research on those unelucidated cases led to the identification of pathogenic large deletions within the DFNB1 locus, which however keep intact the structure of the *GJB2* gene. They can be classified in two different types: those that truncate the neighboring *GJB6* gene and those that also keep intact its structure.

**Figure 3:**
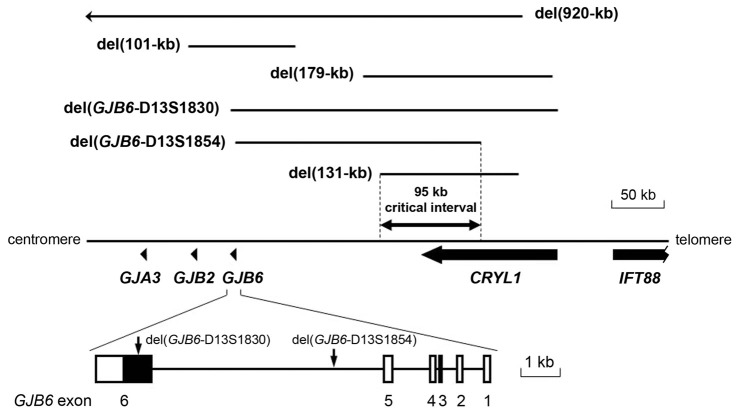
Large deletions on chromosome 13q12 that are responsible for DFNB1 hearing impairment (HI). The proximal breakpoint of del131-kb and the distal breakpoint of del(*GJB6*-D13S1854) delimit the 95.4-kb sequence stretch where the cis-acting element regulating the expression of *GJB2* must be situated. The two proximal breakpoints of deletions del(*GJB6*-D13S1830) and del(*GJB6*-D13S1854; vertical arrows) lie within *GJB6*.

Two deletions were reported to truncate the *GJB6* gene, while keeping *GJB2* intact. The first identified was del(*GJB6*-D13S1830), which removes a 309-kb interval that includes the first five exons of *GJB6* and the whole *CRYL1* gene (Table 1, Figure 3; Lerer et al., 2001; del Castillo et al., 2002, 2003; Pallares-Ruiz et al., 2002). Few years later, another deletion of the same type was characterized. This deletion, named del(*GJB6*-D13S1854), removes a 232-kb interval, with the proximal breakpoint within *GJB6* intron 5, and the distal breakpoint in intron 4 of *CRYL1* (Table 1, Figure 3; del Castillo et al., 2005).

These findings suggested that mutations in *GJB2* and *GJB6* could follow a digenic pattern of inheritance. Results from some studies in humans and rodents fitted well into this hypothesis. First, Cx26 and Cx30 share the same spatial pattern of expression within the cochlea, with apparent co-localization, and can form heteromeric connexons and heterotypic gap-junction channels (Dahl et al., 1996; Lautermann et al., 1998; Forge et al., 2003a,b), although these conclusions have been partly challenged by a recent study using super-resolution structured illumination fluorescence microscopy (Liu et al., 2016). Second, some mutant Cx26 subunits involved in autosomal dominant NSHI are able to exert dominant negative effects on the activity of wild-type Cx30 (Forge et al., 2003b; Marziano et al., 2003). Third, a mutation in *GJB6* was found in a case of autosomal dominant NSHI (Grifa et al., 1999). Finally, *Gjb6* knock-out mice were generated by replacing the complete *Gjb6* coding sequence with a cassette containing the *lacZ* reporter and the *neo* selection gene (Teubner et al., 2003). These mice, termed *Gjb6*^tm1Kwi/tm1Kwi^, have severe deafness and show a complete lack of endocochlear potential (Teubner et al., 2003) due to disruption of the endothelial barrier of capillaries embedded within the stria vascularis (Cohen-Salmon et al., 2007). Furthermore, intercellular transfer of metabolites such as glucose among supporting cells in the organ of Corti of *Gjb6*^tm1Kwi/tm1Kwi^ mice is reduced, though electrical coupling is preserved (Chang et al., 2008). Finally, *Gjb2*^+/−^ *Gjb6*^+/tm1Kwi^ double heterozygous mice have moderate HI and a significantly reduced endocochlear potential (Michel et al., 2003; Mei et al., 2017), although this is in contrast with the severe or profound HI observed in a majority of human double heterozygotes carrying del(*GJB6*-D13S1830) and a pathogenic *GJB2* mutation (Snoeckx et al., 2005). A strong support for the hypothesis of the digenic inheritance would have come from the finding of truncating point mutations in *GJB6* in cases of human autosomal recessive NSHI, but this type of mutations have not been reported to date.

An alternative explanation hypothesized that *GJB2* expression in the cochlea would require a cis-acting regulatory element, which would lie within the interval that is removed by the deletions, and whose absence would abolish the expression of the structurally normal *GJB2* allele that is located downstream. In fact, Cx26 expression in the ductal epithelium of sweat glands was shown to be dramatically reduced in a double heterozygote for *GJB2* c.35delG and del(*GJB6*-D13S1830; Common et al., 2005). In other studies, qualitative allele-specific RT-PCR was used to investigate *GJB2* expression in buccal epithelium cells. In three double heterozygotes for del(*GJB6*-D13S1830) and a *GJB2* mutation), no expression could be detected from the wild-type *GJB2* allele that was in cis with del(*GJB6*-D13S1830; Rodriguez-Paris and Schrijver, 2009). Similar results were obtained when studying three double heterozygous subjects for del(*GJB6*-D13S1854) and a *GJB2* mutation (Rodriguez-Paris et al., 2011).

The hypothesis of the cis-acting regulatory element received further support from the identification and characterization of the second type of DFNB1 deletions, i.e., those keeping the structure of both *GJB2* and *GJB6* intact (Wilch et al., 2006, 2010; Tayoun et al., 2016). The first reported deletion of this type was del(ch13:20,939,344-21,070,698; Wilch et al., 2006, 2010). The deletion interval was 131-kb long. Its proximal breakpoint was located between *GJB6* (more than 100 kb upstream of its 5′ end) and *CRYL1* (Wilch et al., 2010; Table 1, Figure 3). It was found in a family whose affected subjects were double heterozygous, the other allele carrying the c.35delG *GJB2* mutation. Interestingly, qualitative allele-specific RT-PCR, which was performed on buccal epithelium cells from these subjects, revealed a dramatic reduction of the expression from the wild-type *GJB2* and *GJB6* alleles in cis with the deletion. In addition to supporting the existence of the cis-acting regulatory element, these results suggest that it could be implicated in the co-regulation of the expression of *GJB2* and *GJB6*. A second deletion of the same type has been reported recently (Tayoun et al., 2016). It removes a 179-kb interval, with the proximal breakpoint between *GJB6* and *CRYL1*. It was found in a subject with moderate NSHI carrying the p.(Val37Ile) in the other allele.

A re-examination of the mice models also supports the hypothesis of the cis-acting regulatory element. The *Gjb6*^tm1Kwi^ mouse is not only a *Gjb6* knockout, as the engineered mutation also decreases the transcription of the contiguous *Gjb2* gene (Ortolano et al., 2008; Lynn et al., 2011), a polar effect that is likely due to the insertion of the long *lacZ-neo* cassette. Boulay et al. (2013) recently generated an independent *Gjb6* knock-out strain (termed *Gjb6*^Δ/Δ^) that carries a complete deletion of the *Gjb6* coding sequence, but no inserted material, aside from a single *loxP* site, which minimizes the polar effect on *Gjb2*. Indeed, while in the two *Gjb6* knock-out strains cochlear *Gjb2* mRNA and Cx26 protein levels are reduced compared to wild-type controls, *Gjb6*^Δ/Δ^ mice have *Gjb2* mRNA levels similar to those of *Gjb6*^+/tm1Kwi^ heterozygotes (which are not deaf) and five times the amount of Cx26 shown by their *Gjb6*^tm1Kwi/tm1Kwi^ counterparts (Boulay et al., 2013). Critically, *Gjb6*^Δ/Δ^ mice have no HI whatever (Boulay et al., 2013), implying that the impaired hearing observed in *Gjb2*^+/−^ *Gjb6*^+/tm1Kwi^ double heterozygotes is solely caused by the reduced levels of Cx26.

Altogether, currently available evidences from human subjects and mice models strongly support the existence of the regulatory element, but it still remains to be identified. It should lie within the critical 95.4 kb interval that has been established by overlapping all deletion intervals that are known to date (Figure 3).

### Mutations That Affect RNA Splicing

Given the simple structure of *GJB2* (only one intron), few splice-site mutations causing HI have been reported to date in this gene (Denoyelle et al., 1999; Green et al., 1999; Mani et al., 2009; Gandía et al., 2013). Since *GJB2* exon 2 contains the entire coding region, these splice-site mutations have no effect on protein coding.

Mutation c.-23+1G>A affects the donor splice site of intron 1. In a lymphoblastoid cell line derived from a heterozygous individual, cDNA sequencing did not reveal any transcript from the allele with this mutation. It was concluded that either the c.-23+1G>A allele was not transcribed or that the transcript was quickly degraded (Shahin et al., 2002).

Mutation c.-22-2A>C abolishes the acceptor splice site of intron 1. It was first reported in three Spanish siblings who were compound heterozygous for c.-22-2A>C and c.35delG, and presented with mild postlingual HI (Gandía et al., 2013). Later on, this same genotype was reported in an Italian subject with moderate HI (Stanghellini et al., 2014). The effects of the mutation were investigated by RT-PCR on RNA extracted from saliva from one of the siblings of the Spanish family. The acceptor splice site was actually abolished, and so normally processed transcripts from this allele were absent. However, the RT-PCR assays detected residual amounts of an abnormally processed transcript. It was generated by using an alternative acceptor splice site, which resulted in the insertion of a 38-bp intronic sequence into the 5′-UTR, but keeping an intact coding region. Therefore, Cx26 could still be translated from this transcript, although its residual amounts are likely to be insufficient to support a normal function, and this could explain the mild phenotype observed in subjects with this mutation (Gandía et al., 2013).

Given the late onset of the mild HI associated with c.-22-2A>C, it was hypothesized that this mutation could contribute to age-related hearing loss. The presence of the mutation was investigated in a large cohort of Italian subjects with age-related hearing loss and ethnically-matched control subjects with normal hearing (Rubinato et al., 2016). The mutation was found in homozygous, heterozygous, or compound heterozygous state with other known pathogenic missense mutations. There was no significant difference in allelic or genotypic frequencies between patient and control groups (Rubinato et al., 2016). Taking everything into consideration, it seems that c.-22-2A>C would be a hypomorphic recessive allele, like p.(Met34Thr) and p.(Val37Ile), with mild phenotypic effects or no effect at all, depending on the pathogenic potential of the accompanying allele.

Finally, exonic mutation c.-23G>T, lying adjacent to the splice donor site of intron 1, was found in the compound heterozygous state with p.(Trp24*) in an Indian subject with severe to profound NSHI (Mani et al., 2009). Although in silico analysis predicted that it could abolish the use of that donor site, this hypothesis still needs experimental confirmation.

### Mutations in the *GJB2* Coding Region

**Figure 4:**
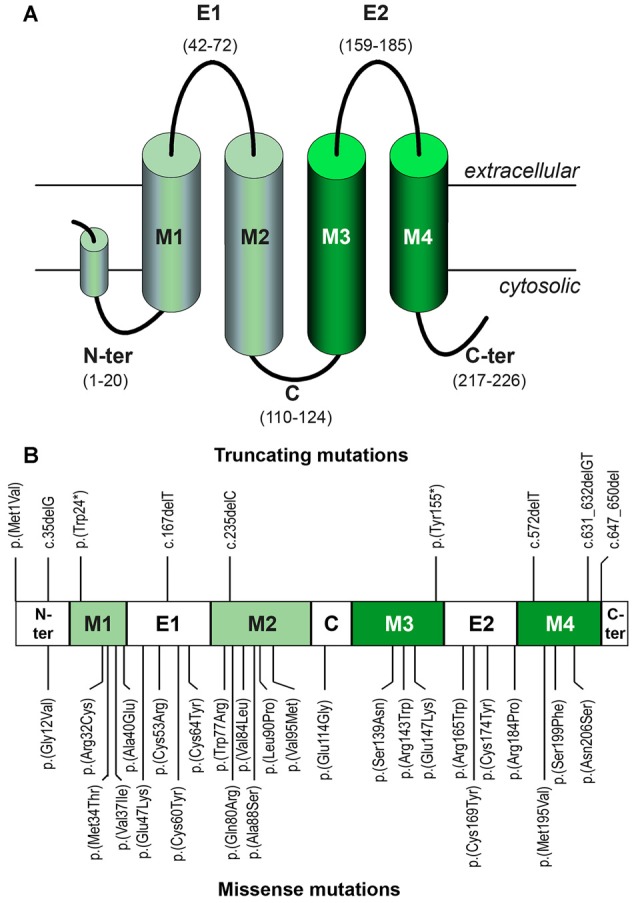
**(A)** Topological organization of the Cx26 monomer with its different structural elements. Helices are represented as cylinders. Helices in light green line the hydrophilic pore of the channel, whereas helices in dark green are exposed to the hydrophobic environment of the lipid bilayer. Note the projection into the cytosol of helices M2 and M3. **(B)** Location of the truncating and missense mutations mentioned in the text within the different structural elements.

A Cx26 monomer is a 226-amino acid long polypeptide that consists of four transmembrane helices (TM1–TM4) linked by two extracellular loops (E1 and E2) and one shorter intracellular loop, with N- and C-termini on the cytosolic side of the membrane (Figure 4A). Newly-synthesized Cx26 monomers undergo conformational maturation and assembly into connexons when moving along the secretory pathway for delivery at the plasma membrane. Once there, Cx26 connexons may remain as so-called hemichannels, allowing for transport of diverse small molecules (less than 1 kDa) between the cytosol and the extracellular space. Alternatively, a connexon may contact, in a head to head disposition, another connexon on the plasma membrane of an adjacent cell to form an intercellular channel. The juxtaposition of hundreds of intercellular channels in plaques forms gap junctions (Kumar and Gilula, 1996), directly linking the cytoplasms of the two adjacent cells and creating a functional syncytium. Mutations in the *GJB2* coding sequence may disrupt any of these maturation processes or they may interfere with hemichannel or intercellular channel function.

#### Mutations Truncating Cx26

Many pathogenic *GJB2* mutations generate premature stop codons, either directly (nonsense mutations; e.g., p.(Trp24*)) or because small insertions, duplications or deletions cause frameshifts (e.g., c.35delG, also known as p.(Gly12Valfs*2)). As the coding sequence of the *GJB2* gene is completely contained in the last exon, mRNAs harboring premature stop codons are expected to escape the nonsense-mediated decay surveillance pathway (reviewed in Lykke-Andersen and Jensen, 2015). Thus, truncated proteins will actually be synthesized, as shown in HeLa cell assays of the c.235delC (p.(Leu79Cysfs*3)), p.(Tyr155*), c.572delT (p.(Phe191Serfs*5)) and c.631-632delGT (p.(Cys211Leufs*5)) mutations (Figure 4B; Choung et al., 2002; Xiao et al., 2011). Nearly all of the known truncating mutations (such as c.35delG (Denoyelle et al., 1997), c.167delT (Zelante et al., 1997), p.(Trp77*) (Kelsell et al., 1997) and c.235delC (Fuse et al., 1999)) result in proteins lacking one or several of the transmembrane segments and intervening loops, which hampers Cx26 folding and oligomerization, resulting in retention at the endoplasmic reticulum (ER) and ultimately causing a total loss of function. This is the case even with just a partial truncation of the TM4 helix and loss of the C-terminal stretch (mutation c.631-632delGT, p.(Cys211Leufs*5); Lim et al., 2003; Xiao et al., 2011). Interestingly, ER retention of the truncated subunits may induce in some cases the unfolded protein response (reviewed in Lindholm et al., 2017) which in turn may eventually lead to apoptosis of cells expressing those *GJB2* mutant alleles, though this hypothesis is yet to be experimentally tested. Only one frameshift mutation in which truncation does not affect the transmembrane helices has been reported so far: c.647-650del (p.(Arg216Ilefs*7); Figure 4B). Although it is a pathogenic mutation (Prasad et al., 2000), its effects have not been functionally assayed yet. It is thus uncertain whether truncation of the C-terminus only would also result in ER retention or it would simply interfere with channel function, since it is believed that the C-terminal hydrophilic stretch, whose crystal structure is not yet solved, may participate in chemical gating or complete pore closure (Maeda et al., 2009).

The p.(Met1Val) mutation (Estivill et al., 1998), which results in no Cx26 protein synthesis at all (p.0 null allele; Thönnissen et al., 2002), although not properly a truncating mutation, should also be included in this category.

#### Missense Mutations

All the remaining DFNB1 pathogenic mutations result in amino acid substitutions. These mutations fall in two categories: those affecting residues involved in folding, oligomerization or structural stability of Cx26 and those that impair one or several aspects of Cx26 function, although any particular mutation may have several different effects. The landmark publication of the crystal structure of a Cx26 connexon (Maeda et al., 2009) provided a framework for integrating at the molecular level data obtained in diverse functional assays of these mutations.

##### Mutations affecting the structure of the Cx26 monomer

In the solved crystal structure, the monomer forms a compact bundle of four helices in which adjacent helices are antiparallel. Helices TM1 and TM2 from each monomer face the interior of the connexon channel, while helices TM3 and TM4 face the hydrophobic environment of the membrane. The N-terminus forms a short helix that is also embedded in the lipid bilayer (Figure 4A). Maeda et al. (2009) identified four groups of residues—two hydrophilic and two hydrophobic cores—that stabilized the structure of the monomer by means of intramolecular interactions (salt bridges or hydrogen bonds) among them. It is reckoned that most amino acid substitutions affecting such residues would result in misfolding and retention of the mutant polypeptide in the ER. Mutations at those residues include: p.(Arg32Cys; Prasad et al., 2000), p.(Arg32His; Mustapha et al., 2001), p.(Arg32Leu; Wu et al., 2002), p.(Gln80Arg; Uyguner et al., 2003), p.(Glu147Lys; Frei et al., 2004), p.(Ser199Phe; Green et al., 1999), p.(Arg143Trp; Brobby et al., 1998), p.(Asn206Ser; Marlin et al., 2001), p.(Asn206Thr; Wattanasirichaigoon et al., 2004), p.(Ser139Asn; Marlin et al., 2001), p.(Ala40Glu; Feldmann et al., 2004a), p.(Val43Met; Hwa et al., 2003), p.(Trp77Arg; Carrasquillo et al., 1997) and p.(Met195Val; Tsukada et al., 2010). Indeed, intracellular retention of the mutant polypeptide in expression experiments performed in transfected cells has been demonstrated for p.(Arg32His; Xiao et al., 2011), p.(Trp77Arg; Martin et al., 1999; Bruzzone et al., 2003), p.(Met195Val; Kim et al., 2016) and p.(Ser199Phe; Ambrosi et al., 2013). However, not all mutations at those residues cause intracellular retention, as shown for p.(Arg143Trp; Wang et al., 2003) and p.(Asn206Ser; Ambrosi et al., 2013). Apparently, those two particular amino acid substitutions can be accommodated within the monomer structure without total disruption of the intramolecular-stabilizing interactions.

Additional stabilization of the monomer is provided by three intramolecular disulfide bonds (Cys53-Cys180, Cys60-Cys174 and Cys64-Cys169) connecting the E1 and E2 loops (Maeda et al., 2009); indeed, formation of those disulfide bonds is an essential part of the Cx26 conformational maturation process. Unsurprisingly, all known mutations replacing any of these cysteine residues result in DFNB1 NSHI, as they probably result in misfolding and intracellular retention of the mutant polypeptides, though to our knowledge none have been investigated in functional assays. Examples include p.(Cys53Arg; Dahl et al., 2001), p.(Cys60Tyr; Taniguchi et al., 2015), p.(Cys64Tyr; Putcha et al., 2007), p.(Cys169Tyr; Azaiez et al., 2004) and p.(Cys174Arg; Gardner et al., 2006).

##### Mutations affecting connexon assembly and oligomerization

Another set of residues participates in intermolecular interactions within the connexon, influencing both correct folding and oligomerization. Most of the mutations affecting those residues are involved in dominantly inherited disorders, because the effect of the amino acid substitution on intermonomer contacts impairs connexon assembly with wild-type Cx26 subunits (Rouan et al., 2001). However, missense mutations at these residues may also result in DFNB1 NSHI, as it happens with p.(Arg184Pro; Denoyelle et al., 1997). Thus, p.(Arg184Pro)-Cx26 is completely incapable of oligomerization in HeLa cell assays (Thönnissen et al., 2002) and so unable to interact with other connexin subunits, which explains why the mutation has no dominant negative effect on wild-type Cx26.

##### Mutations affecting the hydrophilic channel pore

Missense mutations may also target residues of the hydrophilic channel of the connexon. This channel is lined on the cytosolic end by the protrusions of helices TM2 and TM3 of each monomer, with net positive charge, and on the extracellular end by the residues in the N-terminal half of the E1 loop (Figure 4A). The N-terminus helix and the TM1 helix create a funnel in the center of the channel that is involved in voltage-sensitive channel gating (Maeda et al., 2009), with closure due to inside positive potential (Verselis et al., 1994). DFNB1 mutations at these residues, such as p.(Gly12Val; Rabionet et al., 2000), p.(Met34Thr; Kelsell et al., 1997), p.(Val37Ile; Abe et al., 2000) and p.(Arg143Trp), reduce channel permeability (Martin et al., 1999; Thönnissen et al., 2002; Bruzzone et al., 2003; Bicego et al., 2006; Zonta et al., 2014a; García et al., 2015). The replacement of Met-34 with Thr and of Val-37 with Ile have only slight structural effects and thus p.(Met34Thr) and p.(Val37Ile) have weak pathogenic potentials. It must be remembered, though, that the specific amino acid substitution in each case may have very different functional consequences. For example, both p.(Gly12Val)-Cx26 and p.(Gly12Arg)-Cx26 have altered channel permeabilities. However, whereas p.(Gly12Val) only causes DFNB1 NSHI, p.(Gly12Arg) underlies KID syndrome due to a gain of function mechanism in which the mutant subunit becomes able to oligomerize with Cx43 (García et al., 2015). Finally, mutations that kink transmembrane helix TM2 by introducing proline residues alter the orientation of its cytosolic protrusion, which impairs channel permeability. The best known example is p.(Leu90Pro; Denoyelle et al., 1999; D’Andrea et al., 2002; Thönnissen et al., 2002; Bruzzone et al., 2003).

##### Mutations affecting the function of Cx26 hemichannels

Cx26 hemichannels and gap-junction intercellular channels play distinct physiological roles in cochlear cells. Cx26 hemichannels mediate paracrine and autocrine signaling that is essential for the acquisition of hearing during cochlear development and in the function of the mature cochlea. One of the crucial morphogenetic events in cochlear development is the acquisition of Ca^2+^ signaling in all cell types (recently reviewed by Mammano and Bortolozzi, 2017). In supporting cells, developmental Ca^2+^ signaling is mediated by ATP-induced spontaneous oscillations in the cytosolic concentration of free Ca^2+^, which in turn propagate intercellular Ca^2+^ waves. The underlying mechanism is as follows: ATP binding to plasma membrane P_2_Y receptors provokes phospholipase C-dependent synthesis of inositol 1,4,5-trisphosphate (IP_3_), which directs Ca^2+^ release from ER stores. The increase in concentration of intracellular free Ca^2+^ opens Cx26 hemichannels in the apical plasma membrane, which release cytosolic ATP to the endolymph, thereby propagating the Ca^2+^ oscillation to neighboring cells, which helps coordinate cellular activity (Beltramello et al., 2005; Piazza et al., 2007; Anselmi et al., 2008). In mice, these intercellular Ca^2+^ waves control Cx26 and Cx30 expression at the transcriptional level (Rodriguez et al., 2012) and they participate in the final development of cochlear structures, such as the formation of the mature inner sulcus by crenation (osmotic shrinkage of cells) and the functional maturation of inner and outer hair cells (Zhu et al., 2013; Ceriani et al., 2016; Johnson et al., 2017). In fact, supporting cells in the greater epithelial ridge (GER, the precursor of the mature inner sulcus) spontaneously release ATP through connexin hemichannels before hearing onset by the mechanism described above. This spontaneous ATP release coordinates cochlear morphogenetic events with auditory pathway maturation. Autocrine ATP signaling in GER supporting cells triggers both Cl^−^ efflux, which induces crenation, and K^+^ efflux, which causes depolarization of inner hair cells and their subsequent exocytosis of glutamate, leading to the firing of action potentials by spiral ganglion neurons (Tritsch and Bergles, 2010; Wang et al., 2015). Such action potentials evoke coordinated bursts of activity from central auditory neurons in a precise, stereotyped pattern, which is believed to help consolidate synapses in developing circuits all along the auditory pathway (Tritsch et al., 2010). The spontaneous ATP release by supporting cells ceases at hearing onset. A similar role in ATP paracrine signaling in the adult cochlea was also proposed for Cx26 hemichannels (Anselmi et al., 2008), including modulation of outer hair cell electromotility (Zhao et al., 2005), but further experimentation showed that ATP release induced by Ca^2+^ signaling in the mature cochlea is predominantly dependent on pannexin1 and not Cx26 (Chen et al., 2015). Some DFNB1 missense mutations interfere with this hemichannel function, either by reducing the stability of hemichannels at the plasma membrane (p.(Asn206Ser); Ambrosi et al., 2013) or by replacing the Glu-47 residue that is critical for hemichannel closure at high extracellular Ca^2+^ concentration (p.(Glu47Lys); Marlin et al., 2001; Zonta et al., 2014b).

Cx26 hemichannels may also play a second, intriguing role in cochlear physiology. Recently, Cx26 hemichannels have been implicated in CO_2_ chemosensitivity in the central nervous system (Huckstepp et al., 2010a,b). Indeed, CO_2_ directly binds Cx26 at the beginning of the M3 helix, probably by carbamylation of Lys-125 (Figure 4), which induces opening of the Cx26 hemichannel and subsequent ATP release (Meigh et al., 2013). Auditory transduction involves high energy consumption in the cochlea and concomitant CO_2_ production, and thus CO_2_ may evoke Cx26 hemichannel-dependent ATP signaling for coordination of supporting cell activity. At least two DFNB1 mutations interfere with CO_2_-mediated hemichannel opening, which would impair signaling: p.(Met34Thr), which reduces the ability of the hemichannel to open in response to CO_2_, and p.(Ala88Ser; Frei et al., 2002), with a reduced affinity for CO_2_ (de Wolf et al., 2016).

##### Mutations affecting the function of Cx26 gap-junction channels

Auditory transduction depends on three related homeostatic processes: (1) maintenance of extracellular fluid (endolymph and perilymph) composition; (2) preservation of the functionality of hair cells; and (3) production of the endocochlear potential. All three processes rely on extensive gap-junction coupling among nearly all cells in the cochlea, with the exceptions of marginal cells in the stria vascularis and hair cells in the organ of Corti. In humans, recent reports show that Cx26 forms small gap-junction plaques that are clearly separate from, but closely associated to, the larger Cx30 plaques all along the cochlea (Liu et al., 2016, 2017), suggesting that each class of plaque would entirely consist of a single connexin subunit (i.e., each plaque would consist of identical homomeric connexons). How this spatial organization is achieved and what are its pathophysiological implications remain to be determined.

It has been proposed that Cx26 gap junctions participate in the organ of Corti in spatial buffering (also termed “sinking”) of excitotoxic substances—K^+^ and glutamate. Indeed, it was hypothesized that K^+^ was dispersed away from the hair cells and transported back to the endolymph by a gap-junction mediated pathway (Santos-Sacchi and Dallos, 1983; Kikuchi et al., 1995). Later on, the hair cell degeneration observed in one *Gjb2* knockout mouse (Cohen-Salmon et al., 2002) suggested that the major mechanism underlying DFNB1 NSHI might be the loss of such pathway, but recent experiments in mice showing that Cx26 is dispensable for spatial buffering have ruled out this hypothesis (reviewed in Zhao, 2017).

In contrast, Cx26 gap junctions do have an important role in the establishment and maintenance of the endocochlear potential by the stria vascularis, a two-layered epithelium in the lateral wall of the cochlea that generates the endocochlear potential by active transport of K^+^ from perilymph into endolymph (Wangemann et al., 1995; Takeuchi et al., 2000; Marcus et al., 2002; Lang et al., 2007). Within the stria, Cx26 and Cx30 gap junctions convey K^+^ ions actively taken up from the perilymph by fibrocytes and basal cells to intermediate cells which release K^+^ into the intrastrial space. The effects of Cx26 ablation in the stria vascularis have not been explored directly, as mouse models with conditional KO of *Gjb2* in this structure have not been generated to date. However, some insights have been provided by Cx30 KO models. *Gjb6*^tm1Kwi/tm1Kwi^ mice (with critically decreased *Gjb2* expression and no Cx30) show no endocochlear potential (Teubner et al., 2003), *Gjb2*^+/−^ *Gjb6*^+/tm1Kwi^ double heterozygous mice (also with reduced *Gjb2* expression from the *Gjb6*^+/tm1Kwi^ allele) have a significantly reduced endocochlear potential (Michel et al., 2003; Mei et al., 2017) and *Gjb6*^Δ/Δ^ mice are not deaf, indicating a normal endocochlear potential (see “Deletions Removing a Distal Enhancer” section). There are two alternative explanations for these results as regards endocochlear potential generation: either Cx26 is essential and Cx30 is dispensable or each of the two connexins may be able to compensate for the lack of the other. These hypotheses must be tested experimentally.

In addition to these homeostatic roles, recent research has unveiled two roles of Cx26 gap junctions in cochlear development. Coordination of gene expression among cells in the developing cochlea is achieved by gap-junction-mediated transfer of microRNAs (miRNAs) and specific second messengers (such as IP_3_). Interestingly, in the inner ear, only Cx26 gap junctions are permeable to miRNAs (Zhu et al., 2015). Disruption of Cx26 gap junctions in mice blocks miRNA intercellular transfer and results in aberrant organ of Corti development that causes deafness (Kudo et al., 2003; Zhu et al., 2015; Zong et al., 2016). To date, disruption of miRNA permeability has only been demonstrated for the PPK-D mutation p.(Arg75Trp; Zong et al., 2016), but any mutations that reduce channel permeability may interfere with miRNA transfer. Of particular interest are mutations located in the extracellular loops that do not affect connexon intracellular sorting and result in constricted channel pores, such as p.(Arg165Trp; Rickard et al., 2001; Xiao et al., 2011). Future research should address the effects of any mutations on miRNA transfer.

As regards intercellular traffic of second messengers, it is well established that IP_3_ is one of the major players in inner ear morphogenesis, participating in the gap-junction-mediated propagation of intercellular Ca^2+^ waves between coupled cells (Mammano and Bortolozzi, 2017). Among other processes, IP_3_ transfer through gap junctions propagates the signal for cytochrome *C*-dependent apoptosis from the triggering cell to cells within the cell-death spreading zone, a crucial event in inner ear morphogenesis (Decrock et al., 2012). Mutation p.(Val84Leu; Kelley et al., 1998) specifically impairs Cx26-gap junction IP_3_ permeability (Beltramello et al., 2005; Decrock et al., 2012), while being completely undistinguishable from wild-type Cx26 in all other aspects tested in functional assays (Bruzzone et al., 2003; Wang et al., 2003). Impairment of IP_3_ permeability likely happens in other mutations that disrupt gap-junction channel transfer of most substances, as demonstrated for p.(Val95Met; Kelley et al., 1998; Beltramello et al., 2005). However, it seems that impairment of IP_3_ permeability alone is sufficient to cause HI, underscoring the importance of this particular role of Cx26.

##### Harmless variants

The last group of missense variants in *GJB2* does not affect any residues known to be critical for the structure of Cx26. Given the available genetic data, they are considered harmless variants, among them p.(Val27Ile; Kelley et al., 1998), p.(Phe83Leu; Scott et al., 1998), p.(Arg127His; Estivill et al., 1998), p.(Val153Ile; Rickard et al., 2001) and p.(Ile203Thr; Kudo et al., 2000). However, caution is needed when analyzing those genetic data, as indicated by the case of the p.(Glu114Gly) mutation (Fuse et al., 1999). p.(Glu114Gly) nearly always appears in cis with p.(Val27Ile) in a complex allele, as indicated by the notation [p.(Val27Ile); p.(Glu114Gly)]. Variant p.(Glu114Gly) has usually been considered harmless because [p.(Val27Ile); p.(Glu114Gly)] /[p.(Val27Ile); p.(Glu114Gly)] homozygotes had been observed in a large number of normal hearing control individuals (e.g., 18.7% of controls from the Korean population; Choi et al., 2011). However, functional assays of p.(Glu114Gly) alone indicate defective gap junction channel and hemichannel functions that are largely compensated in the complex allele [p.(Val27Ile); p.(Glu114Gly)] (Choi et al., 2011). Thus, p.(Glu114Gly) might underlie DFNB1 HI in those rare cases in which this mutation does not present in cis with p.(Val27Ile).

Overall, accumulating evidence on Cx26 mutations in the *GJB2* coding sequence paints a very complex picture in which particular mutations may have several very different pathological effects, according to the specific Cx26 structural or functional defect(s) that they cause. Truncating mutations, by interfering with most of Cx26 roles in the ear, are supposed to have the most dramatic effects, but even relatively subtle defects that only impair a single function (e.g., p.(Val84Leu)) result in HI. Future research should strive to understand better the pathogenic processes caused by the most common mutations, including: (1) agreeing on a standard set of functional assays that addresses as many Cx26 functional aspects as possible; and (2) paying particular attention to inner ear developmental defects.

### Mutation Screening for the Genetic Diagnosis of DFNB1 HI

Genetic diagnosis of inherited NSHI is complicated, in terms of cost-effectiveness, by the extreme genetic heterogeneity of this condition. So the high prevalence of DFNB1 HI in most populations (Chan and Chang, 2014), together with the simple structure of the *GJB2* gene, which facilitated the design and application of different molecular tests, made screening for *GJB2* mutations the mainstay of genetic testing of inherited NSHI.

Strategies for DFNB1 genetic testing have evolved over the years, following the technical advances of Human Molecular Genetics. As specific *GJB2* mutations are overrepresented in different populations and ethnicities (Chan and Chang, 2014), earliest strategies were based on the detection of some of those mutations through specific tests, eventually followed by Sanger DNA sequencing. Given the increasing multiethnicity of many populations, those approaches were considered insufficient (Chan et al., 2011), and DNA sequencing of the whole coding region and splice sites of *GJB2* was recommended instead (Hoefsloot et al., 2013). This should be complemented by tests detecting deletions in the DFNB1 locus.

The advent of massively-parallel sequencing (MPS) facilitated novel strategies. Currently, the most cost-effective approach is using comprehensive panels of genes involved in inherited HI, which can provide sequencing data as well as detection of copy number variants (Shearer and Smith, 2015). Other laboratories use whole-exome sequencing followed by targeted analysis of genes known to be involved in HI (Zazo-Seco et al., 2017).

## Clinical Features of DFNB1 HI

A summary of the clinical features of DFNB1 HI is presented in Table 2.

**Table 2:** Summary of clinical features of DFNB1 hearing impairment (HI).

Inheritance	Autosomal recessive (simplex and multiplex cases)
Age of onset	Mostly prelingual (not always congenital), but postlingual onset has also been reported
Evolution	Mostly stable, but progression has also been documented
Severity	Mild to profound
	Truncating mutations usually result in more severe phenotypes
Audiogram shape	Flat or down-sloping
Inner ear malformations	Prevalence typically lower than 10%
	Enlarged vestibular aqueduct, Mondini dysplasia
Vestibular function	Apparently normal, but a saccular dysfunction may go undetected (it can be revealed by VEMP or caloric testing)
Cochlear implantation	Good outcomes (improvement of speech perception skills and reading performance).
Phenotype of carriers	Loss of hearing in frequencies higher than 6 kHz
	Earlier onset of age-related hearing loss

### Age of Onset and Severity

DFNB1 HI is mostly prelingual, but postlingual onset has also been reported, usually in association with specific mutations. In most subjects it is stable, but progression has also been documented, usually in cases with late onset. Severity is greatly variable, ranging from mild to profound even among subjects with the same genotype. Audiogram shapes are ordinarily flat or down-sloping.

Although DFNB1 HI is mostly prelingual, it should not be assumed that the onset is actually congenital in all cases. This issue is of concern to programmes for newborn audiological hearing screening, because infants with normal responses at birth could develop a severe HI within the next few months. An early study reported two children who were homozygous for c.35delG in *GJB2*. One of them had normal auditory brainstem responses at birth, but was diagnosed with a profound deafness at age 15 months. The other child had normal hearing at age 5 months, but was diagnosed with severe HI at age 9 months (Green et al., 2000). In another study, nine children who had passed the newborn hearing screening received a diagnosis of NSHI later in life and were shown to carry two *GJB2* mutant alleles. The frequency of non penetrance at birth was estimated to be at least 3.8% (Norris et al., 2006). More recently, 14 deaf children with two *GJB2* mutant alleles were reported to have passed the newborn hearing screening, indicating that the frequency of non penetrance at birth could be at least 6.9% (Minami et al., 2013). In fact, it has been postulated that an early but not always congenital onset of DFNB1 HI could be followed by a quick progression of the hearing loss (Gopalarao et al., 2008). Orzan and Murgia (2007) reported that 26 out of 47 children with profound DFNB1 HI had normal hearing at age 3 months, and this was followed by a sudden and severe deterioration. Also, a c.35delG homozygous boy who was diagnosed with HI at age 8 years suffered a sudden progression at age 23 years (Kokotas et al., 2008). Finally, another study reported an 8-year-old boy who was compound heterozygous for c.35delG and c.299-300delAT, whose late-onset hearing loss had started suddenly and progressed rapidly (Kenna et al., 2010).

Given the large numbers of subjects with a molecular diagnosis of DFNB1 HI, correlations between genotype and severity of the hearing loss have been studied in detail. In the largest cross-sectional study so far, genetic and audiological data were gathered from over 1500 affected subjects from 16 countries (Snoeckx et al., 2005). The severity of the HI was widely diverse even for the same genotype. However, when mutations were classified according to the severity of their molecular effects, some correlations were revealed. Genotypes including two truncating mutations (those mutations leading to premature stop codons) resulted in significantly more severe hearing losses than genotypes containing two non-truncating mutations (those mutations leading to amino acid substitutions; Snoeckx et al., 2005). Truncating/non-truncating genotypes resulted in phenotypes of intermediate severity between those two groups. Interestingly, a few genotypes resulted in HI with specific degrees of severity. Thus, profound HI was observed in the majority of cases with the c.35delG/p.(Arg143Trp) or c.35delG/del(*GJB6*-D13S1830) genotypes (Snoeckx et al., 2005). In contrast, mild or moderate HI was common in subjects carrying p.(Leu90Pro), p.(Met34Thr), or p.(Val37Ile) in combination with a mutation of the truncating type (Snoeckx et al., 2005). Further studies in other cohorts have confirmed these conclusions (Azaiez et al., 2004; Liu et al., 2005; Marlin et al., 2005; Primignani et al., 2009; Bartsch et al., 2010; Chan et al., 2010; Kenna et al., 2010; Tsukada et al., 2010; Burke et al., 2016).

There have been many contradictory reports on the pathogenicity of two missense mutations, p.(Met34Thr) and p.(Val37Ile) (Kelley et al., 1998; Griffith et al., 2000; Kudo et al., 2000; Marlin et al., 2001; Feldmann et al., 2004b). Controversy arose because of their high carrier rates is some populations and because they have been found in hearing-impaired subjects but also in subjects with normal hearing, as part of homozygous or compound heterozygous genotypes, the other allele being a clearly pathogenic mutation. All available data are consistent with the interpretation that these two alleles are hypomorphic, i.e., they have low penetrance and weak pathogenic potential. Accordingly, it is expected that their associated phenotypes range from normal hearing to late onset, progressive, mild to moderate hearing loss (Cucci et al., 2000; Houseman et al., 2001; Dahl et al., 2006; Huculak et al., 2006; Schrijver and Chang, 2006; Pollak et al., 2007; Tsukada et al., 2010; Zhao et al., 2011; Kim et al., 2013; Chai et al., 2015; Huang et al., 2015; Du et al., 2016).

The wide variability in severity of DFNB1 HI could be attributed at least partly to the influence of genetic modifiers. A large whole-genome association study investigated the phenotypic variability of c.35delG homozygotes by grouping cases in two classes according to the severity of the phenotype (mild/moderate HI vs. profound HI; Hilgert et al., 2009). The analysis was performed in two steps. First, a set of 255 samples was investigated by using a pooling-based strategy, followed by individual genotyping of the top 250 most significantly associated SNPs, in the same sample set. In a second step, those SNPs that still had significant *P*-values were genotyped in an independent set of samples. After this replication assay, nine SNPs still had significant *P*-values. Results from this study suggest that the variability in the severity of the HI that is observed in c.35delG homozygotes is not caused by one major genetic modifier, and that those nine SNPs might individually contribute just small modifying effects (Hilgert et al., 2009).

### Temporal Bone

High-resolution computed tomography (CT) scans of the temporal bone have been used to investigate putative inner ear malformations in subjects with DFNB1 HI. Early studies did not reveal temporal bone anomalies (Cohn et al., 1999; Denoyelle et al., 1999). Subsequent studies have coincided in establishing that temporal bone malformations in subjects with DFNB1 HI would not be frequent, their prevalence being typically lower than 10% (Kenna et al., 2001, 2011; Preciado et al., 2004; Azaiez and Smith, 2007; Lee et al., 2009). Findings included unilateral or bilateral enlarged vestibular aqueduct, and bilateral Mondini dysplasia. In contrast with these results, one study found temporal bone anomalies in up to 72% of subjects with DFNB1 HI. These anomalies included hypoplastic cochlea, hypoplastic modiolus, dilated endolymphatic fossa, or enlarged vestibular aqueduct (Propst et al., 2006). This discrepancy has been attributed to differences in the composition of the cohorts of studied subjects (severity of hearing loss, genotypes…) as well as in methodology (radiologic image acquisition and interpretation; Propst et al., 2007; Kenna et al., 2011).

Histopathology of temporal bones in DFNB1 HI has been scarcely explored. In the only report published so far, samples from subjects with congenital profound HI were selected from the archives of a repository of temporal bones for genetic testing. Only one of the selected samples carried two mutant *GJB2* alleles (c.35delG/p.(Glu101Gly) compound heterozygote). Microscopic analysis of cochlear sections from this subject revealed some gross anomalies of the inner ear: agenesis of the stria vascularis, a detached tectorial membrane, and extensive degeneration of hair cells. In contrast, no degeneration of neural structures (spiral ganglion cells and eighth cranial nerve) was observed. Damage to those cochlear structures could be the result of Cx26 deficiency. However, it must be taken into account that the subject had a history of diabetes mellitus, coronary atherosclerosis, hypertension and chronic renal failure, pathologies that might have contributed to those degenerative processes (Jun et al., 2000).

### Vestibular Function

Subjects with DFNB1 HI usually do not complain of vertigo or dizziness, so their vestibular function is not routinely explored, and reports on this issue are sparse. In a study, vestibular-evoked myogenic potentials (VEMP) were absent bilaterally in two subjects carrying two pathogenic *GJB2* mutations. This finding is suggestive of a saccular dysfunction. However, the patients did not report vertigo or dizziness, probably as a consequence of central compensation (Todt et al., 2005). Also, VEMP and caloric responses could not be elicited in 3 out of 5 subjects with two pathogenic *GJB2* mutations (Zagólski, 2007). In another study on seven subjects with DFNB1 HI, vestibular dysfunction was bilateral in one subject and unilateral in four subjects, as revealed by either VEMP recording or the caloric test (Kasai et al., 2010). In a series of 23 subjects with two mutant DFNB1 alleles, only 2 had unilaterally abnormal caloric responses, but 17 showed decreased VEMP amplitudes (Tsukada et al., 2015), which again would suggest a saccular dysfunction. No patient in this series complained of vertigo or dizziness (Tsukada et al., 2015). In another study, a survey was used to assess symptoms of vestibular dysfunction in subjects with two *GJB2* mutant alleles, and 127 out of 235 participants (54%) reported dizziness and vertigo (Dodson et al., 2011).

Taking into account the available data, it seems that vestibular dysfunction may be more common in DFNB1 HI than previously recognized, but its manifestations might be so subtle that they could go easily undetected, unless revealed by VEMP recording. Vestibular testing of larger series of DFNB1 hearing-impaired subjects is needed to clarify this issue.

### Outcome of Cochlear Implantation

Several studies have compared the outcome of cochlear implantation in subjects with DFNB1 HI vs. subjects with non-DFNB1 HI. Speech perception skills improved clearly after implantation in the two groups. In some studies, differences between the two groups were not significant (Green et al., 2002; Bauer et al., 2003; Cullen et al., 2004; Taitelbaum-Swead et al., 2006; Connell et al., 2007), whereas in other studies the DFNB1 HI group showed better outcomes (Fukushima et al., 2002; Matsushiro et al., 2002; Sinnathuray et al., 2004). These differences are likely due to the heterogeneous compositions of the non-DFNB1 HI groups, which include HI of different, sometimes unknown, etiologies. In contrast, reading performance was consistently better in the DFNB1 HI group (Green et al., 2002; Bauer et al., 2003).

### Audiologic Phenotype of Carriers

In agreement with the recessive inheritance pattern of DFNB1 HI, heterozygous carriers of DFNB1 mutations do not show any obvious hearing anomaly. However, some studies have revealed subtle audiological alterations in some subjects. In a cohort of heterozygous carriers for different *GJB2* mutations (c.35delG, p.(Trp77Arg), p.(Val37Ile)), conventional pure-tone audiometry and auditory brainstem responses were normal. However, testing for distortion-product oto-acoustic emissions revealed significantly lower amplitudes in carriers than in non-carrier controls (Engel-Yeger et al., 2002, 2003). These results were not replicated in a different cohort (Groh et al., 2013). Disparities in the results could be atributed in part to the heterogeneous composition of the non-carrier groups. In other studies, pure-tone audiometry revealed significant hearing losses for frequencies higher than 6 kHz in c.35delG heterozygous carriers (Franzé et al., 2005; Groh et al., 2013). Recently, a study on subjects who were heterozygous for the splice-site mutation c.-23+1G>A revealed an earlier onset of age-related hearing loss (at about age 40 years) that in the control group (Barashkov et al., 2014). Studies on these issues are still sparse and some of them provide disparate results, indicating that investigation of larger series of carriers of different mutations is needed to establish firm conclusions.

## Author Contributions

FJC and IC conceived the study, wrote the text and prepared Tables and Figures.

## Conflict of Interest Statement

The authors declare that the research was conducted in the absence of any commercial or financial relationships that could be construed as a potential conflict of interest.

## Footnotes

^1^http://hereditaryhearingloss.org

^2^http://davinci.crg.es/deafness/
